# Infective Juveniles of the Entomopathogenic Nematode *Steinernema scapterisci* Are Preferentially Activated by Cricket Tissue

**DOI:** 10.1371/journal.pone.0169410

**Published:** 2017-01-03

**Authors:** Dihong Lu, Claudia Sepulveda, Adler R. Dillman

**Affiliations:** Department of Nematology, University of California Riverside, Riverside, California, United States of America; The University of Tokyo, JAPAN

## Abstract

Entomopathogenic nematodes are a subgroup of insect-parasitic nematodes that are used in biological control as alternatives or supplements to chemical pesticides. *Steinernema scapterisci* is an unusual member of the entomopathogenic nematode guild for many reasons including that it is promiscuous in its association with bacteria, it can reproduce in the absence of its described bacterial symbiont, and it is known to have a narrow host range. It is a powerful comparative model within the species and could be used to elucidate parasite specialization. Here we describe a new method of efficiently producing large numbers of *S*. *scapterisci* infective juveniles (IJs) in house crickets and for quantifying parasitic activation of the IJs upon exposure to host tissue using morphological features. We found that parasite activation is a temporal process with more IJs activating over time. Furthermore, we found that activated IJs secrete a complex mixture of proteins and that *S*. *scapterisci* IJs preferentially activate upon exposure to cricket tissue, reaffirming the description of *S*. *scapterisci* as a cricket specialist.

## Introduction

Entomopathogenic nematodes (EPNs) are specialized insect parasites that kill their hosts quickly and associate with bacteria as part of their parasitic life style [1, 2]. EPNs have become models of parasite host-seeking behavior [3, 4], parasite neurobiology [5, 6], and eukaryote-bacterial symbioses [7–9]. *Steinernema scapterisci* is an understudied EPN with unusual biology, distinct from other EPNs, and has potential as an important species for comparative studies among EPNs.

The association between parasitic nematode and insect-pathogenic bacteria is one of the defining characteristics of entomopathogeny [10]. The bacteria are thought to be necessary for two reasons; they are highly toxic to insects and are considered to be the pathogenic member of the nematode-bacterium complex [2], and the bacteria seem to be necessary for successful nematode reproduction of most species [2, 11, 12]. Most of the associations between EPNs and their symbiotic bacteria are reported to be monospecific, with each species of nematode only associating with one species of pathogenic bacteria (e.g. [2, 7]). *S*. *scapterisci* is one of the few known exceptions; not only have multiple species of bacteria been isolated from surface-sterilized infective juveniles (IJs) [13], but in contrast to other EPNs, *S*. *scapterisci* appears to be able to reproduce on several different species of bacteria [14–16]. This makes *S*. *scapterisci* an important comparative species for studies of nematode-bacteria symbioses. Furthermore, there is compelling evidence that *S*. *scapterisci* may be highly pathogenic to insect hosts and capable of reproducing *in vivo* without the presence of its described bacterial symbiont, *Xenorhabdus innexi* [12, 17].

*S*. *scapterisci* was first described in 1990 as part of a search for natural enemies of *Scapteriscus* mole crickets, and *S*. *scapterisci* was found to infect and kill mole crickets in their natural environments [18, 19]. *S*. *scapterisci* was then developed as a biological control agent to replace or at least complement chemical pesticides in controlling invasive *Scapteriscus spp*. mole cricket populations. *S*. *scapterisci* has been widely used in biological control in the U.S. and abroad and is one of the most successful EPNs ever used in biological control, reducing pest mole cricket populations to as little as 5% of their pre-treatment population size [20–22]. From an applied perspective *S*. *scapterisci* is a valuable parasite in biological control, and is also one of the few EPNs with a known natural host [23]. The genus *Steinernema* has more than 70 described species, many of which are reported to have broad host ranges [2, 23]. *S*. *scapterisci* is closely related to *S*. *carpocapsae* [24], which is the most well-studied EPN and has the broadest reported host range within the genus, being capable of infecting more than 250 different species of insects across multiple orders [25]. However, unlike its close relative, *S*. *scapterisci* is thought to be a cricket specialist and is less efficient at infecting non-cricket hosts [18, 26]. Specialist parasites are predicted to closely track the evolutionary trajectory of their hosts, have lower virulence, be more speciose, and in EPNs, where they have a mutualistic association with bacteria, this association is predicted to be tighter [27–29]. The biology influencing host-specificity remains unknown among EPNs, but *S*. *scapterisci* is one of the few known specialists within the genus and is an excellent model for studying host specificity and host-parasite interactions in an ecologically relevant way.

Despite the potential of this species as a comparative model, few studies of EPN biology include it. This may be due to *S*. *scapterisci's* specialized biology and host preference for crickets, which make it more difficult to work with than other better-studied EPNs such as *S*. *carpocapsae*. Here we report the development of an improved method for efficient cultivation of *S*. *scapterisci in vivo* using house crickets. We also describe a new morphology-based system to quantify the parasitic activation of IJs upon exposure to insect tissue homogenate. We found that the activated IJs secrete a complex protein mixture that likely plays a role in parasitism. We use this method of activation to study the host specificity of *S*. *scapterisci* and thereby improve its tractability as a comparative model.

## Materials and Methods

### Nematodes and insects

**Fig 1:**
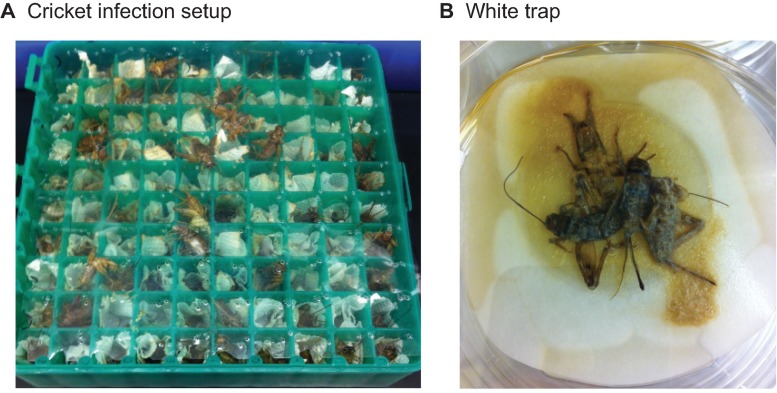
Rearing *S*. *scapterisci* in cricket hosts. **A.** House crickets were anesthetized at 4°C and were then put individually into the wells of a cryo storage box. Each well was lined with a small piece of paper towel loaded with approximately 100 IJs of *S*. *scapterisci* suspended in 100 μl of tap water. **B.** About 3–4 infected crickets were transferred to White traps [31].

Infective juveniles of the *S*. *scapterisci* FL strain were produced by infecting adult *Acheta domesticus* house crickets (~6 week old, mostly adults) (American Cricket Ranch, Lakeside, CA), following a rearing technique modified from Kaya and Stock [30, 31]. Briefly, house crickets were infected by *S*. *scapterisci* IJs in the wells of a plastic cryo storage box with 100 slots designed for 1.5 ml microtubes (e.g., VWR Cat# 40000–322), with the lid removed. A sheet of transparent plastic was cut to fit the top of the box and cover all the wells to prevent crickets from escaping; small holes were punched in the plastic for aeration. Single layers of paper towels (~4 cm x 4 cm) were pushed into the wells of the microtube storage box using a pen to make infection chambers. About 100 IJs of *S*. *scapterisci* suspended in 100 μl of tap water were added to each well lined with a layer of paper towel, which stays moist for at least 2 days and allows nematodes to disperse. Just before infection, the dead and unhealthy house crickets were discarded and healthy adults were anesthetized in a cold room (4°C) for about 10 mins or until most of the crickets had stopped moving. The crickets were then placed individually into the prepared infection chambers (optional: this step can be done in the cold room to prevent crickets from recovering from their chill coma) (Fig 1A). The box was covered by the plastic sheet described above, which was pushed against the wells using a slightly smaller freezer box (e.g., VWR Cat# 82007–162). The boxes were securely bound together by rubber bands or tape. The infection boxes were incubated for 2 days in the dark at 25°C. Dead crickets, presumably infected, were transferred to White traps [31, 32] in which a piece of 5.5 cm filter paper was raised by the lid of a 3.5 cm petri dish in a 10 cm petri dish with a thin layer of tap water (about 2 mm deep) (Fig 1B). About 3–4 infected crickets were placed in the middle of the wet filter paper without directly touching the water (Fig 1B). After 1–2 weeks the IJs gradually migrated into the surrounding water. The IJs were collected and washed with tap water at least 3 times before being stored in tissue culture flasks with vented caps (VWR Cat# 10062–860). Washing large populations of IJs was performed in a glass vacuum filter holder (Fisher Scientific, Cat# 09-753-1C) with two layers of 11 μm nylon net filters (Millipore, NY1104700). Small populations of IJs were washed in 15 ml tubes by centrifugation at 700 rcf for 1 min at room temperature in a swing-bucket rotor. The supernatant was removed after each wash. The density of IJs was controlled to be less than 10 IJs/μl to prevent oxygen depletion. The IJs were stored at 15°C until used. IJs remain largely quiescent and can usually survive for a year or longer under these conditions. For activation experiments, IJs were used within 3 months after collection.

*Galleria mellonella* waxworms used for activation experiments were purchased from CritterGrub (http://www.crittergrub.com/). Once received, waxworms were immediately sorted to remove dead ones, and the healthy waxworms were frozen at -80°C until used. *Acheta domesticus* house crickets (American Cricket Ranch, Lakeside, CA) were immediately sorted to remove dead ones, and the healthy crickets were frozen at -80°C until used. *Scapteriscus borellii* mole crickets used for activation experiments were collected from the Rio Hondo Golf Course (Downey, CA) as previously described [33]. Adult mole crickets were fed a diet of wheat germ (15 g agar, 166 g wheat germ, 900–1000 ml H_2_O) and water *ad libitum* until used. Healthy adult mole crickets were frozen at -80°C prior to use in activation experiments.

### Homogenate production from different insects

We made insect homogenate from insects to test the recovery of IJs in host tissues. To do this, a certain mass of the frozen insects was weighed based on the desired volume of homogenate (e.g., 100 ml of 50% homogenate can be made from 50 g of insects). The insects were ground in liquid nitrogen with a mortar and pestle into a fine powder, which was then transferred to a clean beaker. Phosphate-buffered saline (PBS, 137 mM NaCl, 2.7 mM KCl, 10 mM Na_2_HPO_4_, 1.8 mM KH_2_PO_4_, pH 7.4) was added to suspend the powder to reach the desired final volume. The beaker was loosely covered with plastic wrap and heated in a microwave oven until the suspension started boiling. The suspension was mixed and heated again. The boiling step was repeated 3–4 times in total. The homogenate was cooled to room temperature. The contents were transferred to 15 ml or 50 ml tubes and centrifuged at 3000 rcf for 5–10 min at room temperature. The supernatant with the lipid layer (lipid content was high in *G*. *mellonella* and low in crickets) was collected in new tubes and centrifuged two more times to remove as much solid debris as possible. The final supernatant was mixed vigorously before being aliquoted and frozen at -20°C for future use. The homogenate was supplemented with 1x antibiotics immediately prior to use (penicillin, streptomycin, and neomycin solution; Sigma, P4083-100ML, Caisson Labs, UT). To make 1 ml of crude cricket homogenate, we combined 750 μL of ground cricket and 250 μL of 50% cricket homogenate.

### Small-scale activation of IJs

A method for cultivating nematodes *in vitro* [34] was modified for activating IJs. Sponge foam (Drosophila plugs—Biologix 51–17725) was cut into ~3 mm x 3 mm x10 mm pieces. For a small-scale activation assay, 0.08 g of sponge pieces were repeatedly squeezed in 1 ml of insect homogenate supplemented with 1x antibiotics (Sigma, P4083-100ML) to absorb the homogenate. The homogenate to sponge ratio of 12:1 (volume/weight) was used to obtain a balance between food content and aeration in the sponge matrix [34]. The sponge pieces were then piled in a 35 mm Petri dish. About 20,000 IJs were pelleted by centrifugation at 800 rcf for 1 min at room temperature. The water was removed and replaced with PBS. After being washed three times in PBS, the IJs were resuspended in about 500 μl of total volume and evenly distributed onto the sponge. The petri dish was covered with plastic wrap poked with 10–15 small holes and incubated for 18 hrs at 28°C in the dark. Each activation experiment was repeated at least 3 times.

### Quantification of the number and degree of IJ activation

To recover nematodes from the small-scale activation assays, tap water was added to soak the sponge pieces in the Petri dish for 5 min and the nematode suspension was transferred to a 15 ml tube. The process was repeated two more times and all nematodes were combined in one 15 ml tube. The nematodes were pelleted by centrifugation at 700 rcf for 1 min and washed 3 times with tap water. The supernatant was removed leaving ~1.5 ml of nematode suspension. Aliquots of the nematode suspension were visualized with a compound microscope at the 10 x 40 magnification and scored for activation. Higher magnification was occasionally used when the morphology was unclear. 3–4 aliquots were quantified per treatment meaning that 150–600 nematodes were used for each replicate.

**Fig 2:**
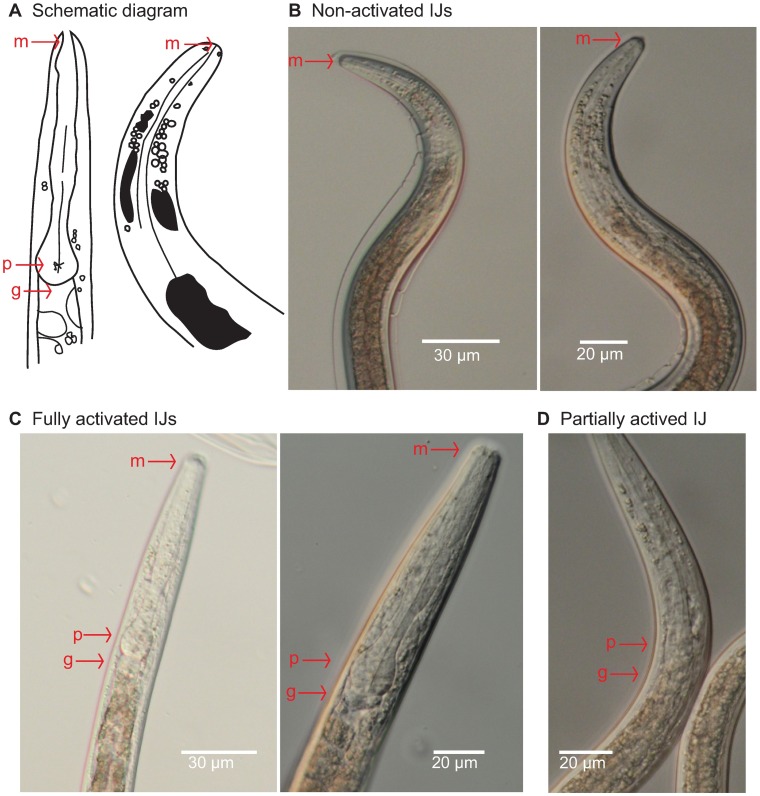
Quantifying IJ activation. The mouth, pharynx, and anterior gut are marked in these images by red arrows and labeled m, p, and g respectively. **A.** Schematic drawing illustrating the morphological features of an activated IJ on the left and a non-activated IJ on the right. In the non-activated IJ the pharynx and anterior gut can be difficult to visualize. **B.** Two pictures showing the opaque morphology of non-activated IJs. The mouth is closed, the anterior gut is closed, and the pharyngeal bulb is not visible. **C.** Two pictures showing the visible morphology of activated IJs. These fully activated IJs have visibly open mouths and stoma. They have expanded pharyngeal bulbs and the anterior gut is open. **D.** This picture shows a nematode scored as partially active because the pharyngeal bulb is only partially expanded.

*S*. *scapterisci* activation was categorized based on 3 morphological features (Fig 2)—(1) the mouth being open or closed, (2) the state of the anterior gut, or the opening of the gut immediately posterior to the pharyngeal bulb, and (3) expansion of pharyngeal bulb and how pronounced it appeared. If an individual nematode had all three characteristics (an open mouth, an open and expanded anterior gut, and an expanded and visible pharyngeal bulb) they were considered fully activated (Fig 2C). If the nematode had a visible pharyngeal bulb, though had not proceeded to a further stage of activation as indicated by the gut and mouth opening, it was considered partially activated (Fig 2D). If the nematode had no visible pharyngeal bulb, it was considered non-activated (Fig 2B). non-activated nematodes were generally still ensheathed, though presence of the L2 cuticle sheath was not used to determine nematode activation. Dead nematodes were not counted. Any nematodes that had developed beyond L3 into L4 or even adulthood were counted as fully activated.

### Time course of IJ activation

IJs were exposed to insect homogenate for different amounts of time: 6, 12, 18, 24, and 30 hours. Though a 3-hour exposure was initially tested, it was too difficult to reliably distinguish morphological characteristics. For the time course we used 50% *Acheta domesticus* homogenate, made as described above.

### Activation using different host homogenates

*S*. *scapterisci* IJs were exposed to three different host homogenates; *Scapteriscus borellii*, *Acheta domesticus*, and *Galleria mellonella*. The homogenates were made in different concentrations (10%, 25%, 50% and crude). We were unable to test crude waxworm homogenate for technical reasons; the crude homogenate was too thick to be separated from nematodes after exposure but we were able to test 10%, 25%, and 50% waxworm homogenate. Each of these activation experiments was collected and observed after 18 hours of exposure to the homogenate. IJ activation was then quantified as described above.

### Large-scale activation of IJs for collecting secreted proteins

About 2.5 x 10^6^ IJs were washed at least 3 times with washing solution (autoclaved 0.8% NaCl solution containing 0.01% Triton X-100 which prevents IJs from sticking to plastic and glass surface) in a glass vacuum filter holder (Fisher Scientific, Cat# 09-753-1C) with two layers of 11 μm nylon net filters (Millipore, NY1104700). The IJ suspension was then transferred to a 15 ml tube and centrifuged at 700 rcf for 0.5–1 min at room temperature. The supernatant was removed leaving about 5 ml of suspended IJs. The IJs were transferred to a 1 L flask containing 8.5 g of autoclaved sponge pieces soaked with 100 ml of 50% house cricket homogenate and 1x antibiotics (Sigma, P4083-100ML). The sponge and IJs were incubated for 12 hrs at 28°C in the dark to activate the IJs. To recover the nematodes after incubation, the sponge pieces were soaked in a 1 L beaker containing about 500 ml of washing solution for 5–10 min, occasionally stirring gently (avoid squeezing the sponge since it could damage nematodes). The nematode suspension was transferred to another beaker and 500 ml of fresh washing solution was added to the sponge. The soaking step was repeated 5–6 times to increase the number of IJs recovered. The nematodes were collected in the glass vacuum filter mentioned above. The solution was removed, leaving only a thin layer of solution covering the nematodes. 100 ml of washing solution was immediately added and the nematodes were resuspended by pipetting. This washing step was repeated at least 10 times to remove contamination. The clean nematodes were resuspended in 105 ml of PBS solution and transferred to a 1 L glass flask (autoclaved) for the collection of secreted products. The flask was incubated in a shaker set to 28°C and 200 rpm for 3 hrs. Then the nematodes were pelleted in 15 ml tubes by centrifugation at 700 rcf for 0.5–1 min at room temperature. The supernatant was filtered through a low protein binding 0.22 μm syringe filter (Fisher Scientific, Cat# 9719001) to remove residual amount of nematodes and bacteria (probably released from activated nematodes) and collected in two 50 ml tubes. The filtered supernatant containing secreted/excreted products (ESPs) was stored at -20°C or directly concentrated in an Amicon Ultra 15 ml centrifugal filter with 3 kDa membranes which retains proteins larger than 3 kDa (Millipore, UFC900308). The centrifugal filter was filled with 15 ml of ESPs and centrifuged in a swing-bucket rotor at 3000 rcf at 4°C for 50–60 min to concentrate the proteins to <1 ml. The flow-through solution was discarded and another 15 ml of ESPs was added to the centrifugal filter. The centrifugation was repeated until the 100 ml of ESPs was concentrated to the dead volume of the filter (~200 μl). The concentrated ESPs were transferred to a 1.5 ml low retention tube (Fisher Scientific, Cat# 21402903). Protein concentration was measured using Bio-Rad protein assay dye reagent (Bio-Rad, 500–0006). The remaining concentrated ESPs were stored at -80°C until use.

### Protein electrophoresis and gel staining

Proteins were denatured by SDS sample buffer (final 1x buffer: 50 mM TrisHCl pH6.8, 2% SDS, 2.5% Ficoll, 0.01% bromophenol blue, and 0.1 M DTT) and heating for 10 min in boiling water before being loaded to the 4–15% Mini-PROTEAN® TGX™ Precast Gels (Biorad, 456–1084). Protein molecular weights were marked by the Precision Plus Protein Dual Color Standards (Biorad, #1610374). The electrophoresis was run at 100 Volts for 60–90 min and the gels were stained by Colloidal Coomassie Blue [35] or by the Pierce Silver Staining kit (Pierce, #24600). Equal percentages of the total volumes of collected proteins from non-activated and activated *S*. *scapterisci* IJs were loaded onto each gel. 10% of protein volumes of activated (total 210 μl x 1.08 μg/μl) and non-activated (total 200 μl x 0.008 μg/μl) IJs were loaded onto the gel for Colloidal Coomassie Blue staining; for the silver staining, the volumes were 1% of the total collected proteins.

### Statistical analysis

Statistical analysis was performed using GraphPad Prism 6.04. Standard statistical tests were used for all experiments, as described in the figure legends. One-way ANOVAs were conducted to compare experimental effects among different exposure times to 50% house cricket homogenate and the effects of the percentage of host homogenate on activation and partial activation. Unpaired t tests were used to compare the experimental effects between two treatments within an experiment (e.g. differences between two time points of the time course activation or differences between two homogenate concentrations within an experiment).

## Results

### Efficient method for rearing *S*. *scapterisci*

Although the technique for producing EPN IJs *in vivo* where insect hosts are infected by IJs in a Petri dish lined with moist filter papers is simple and well-defined for most EPNs [30, 31], it is less convenient and efficient for rearing *S*. *scapterisci*. First, crickets move faster and have smaller contact surface with the filter paper where the IJs are added, and may reduce the infection rate and synchronicity. Second, killed crickets tend to desiccate faster and are often eaten by living crickets. Third, living crickets can easily escape when the Petri dish is opened to add crickets during infection or to retrieve dead crickets after infection.

Our method of infection using a 100-well cryo storage box allowed us to efficiently produce millions of IJs while avoiding unnecessary loss of host crickets due to cannibalism or desiccation (Fig 1). The disadvantages of the conventional Petri dish-based infection method were addressed in the new method. The crickets were confined in the wells lined with paper so that the crickets cannot easily escape; there was larger contact surface where IJs can infect the hosts; living crickets cannot consume the dead ones due to spatial separation and more synchronized infection; and the relatively closed space prevented rapid desiccation of dead crickets. For the experiments reported here we were easily able to produce approximately six million *S*. *scapterisci* IJs from about 200 house crickets infected by 100 IJs/host with minimal effort.

### Infective juvenile activation over time

**Fig 3:**
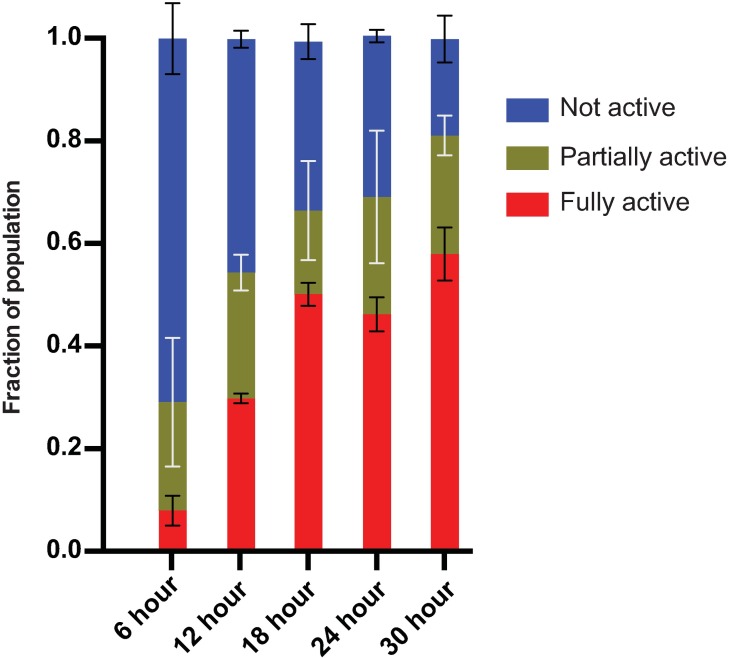
Time course activation of *S*. *scapterisci* IJs. IJs were exposed to 50% house cricket homogenate for different amounts of time and were then quantified for activation. Three biological replicates of triplicate experiments were performed for each time point (n≥249 nematodes per replicate; n≥891 nematodes total per time point) (S1 File). *S*. *scapterisci* IJs activate in a time-dependent manner when exposed to house cricket homogenate (p<0.0001; one-way ANOVA). 12h, 18h, 24h, and 30h exposure led to significantly more activation than 6h exposure (p<0.0001; unpaired t test). 18h, 24h, and 30h exposure led to significantly more activation than 12h (p≤0.0002; unpaired t test). However, there was no significant difference in IJ activation between 18h, 24h, and 30h exposure times (unpaired t test). There we no significant differences in the ratio of partially activated IJs in the time course experiments (one-way ANOVA).

We performed an activation time course, exposing *S*. *scapterisci* IJs to 50% house cricket homogenate for 6, 12, 18, 24 and 30 hours. In order to quantify activation, we used three morphological features: the mouth, anterior gut opening, and the pharyngeal bulb (Fig 2). We scored IJs using three categories; non-activated, partially activated, and fully activated, depending on their morphology as described in the Materials and Methods (Fig 2). Some nematodes developed to L4 and even young adults at later time points, but here they are considered fully activated. We found that *S*. *scapterisci* IJs activated in a time-dependent manner when exposed to house cricket homogenate (p<0.0001) (Fig 3 and S1 File). After 6 hours of exposure, relatively few IJs were fully activated while after 18 hours more than half of the population was fully activated (Fig 3). Comparing exposure times, 12 hr, 18 hr, 24 hr, and 30 hr exposures led to significantly more activation than 6 hr exposure (p<0.0001). 18 hr, 24 hr, and 30 hr exposure led to significantly more activation than 12 hr (p≤0.0002). However, there was no significant difference in IJ activation between 18 hr, 24 hr, and 30 hr exposure times. There were no significant differences in the ratio of partially activated IJs in the time course experiments.

### Protein secretion by activated IJs

**Fig 4:**
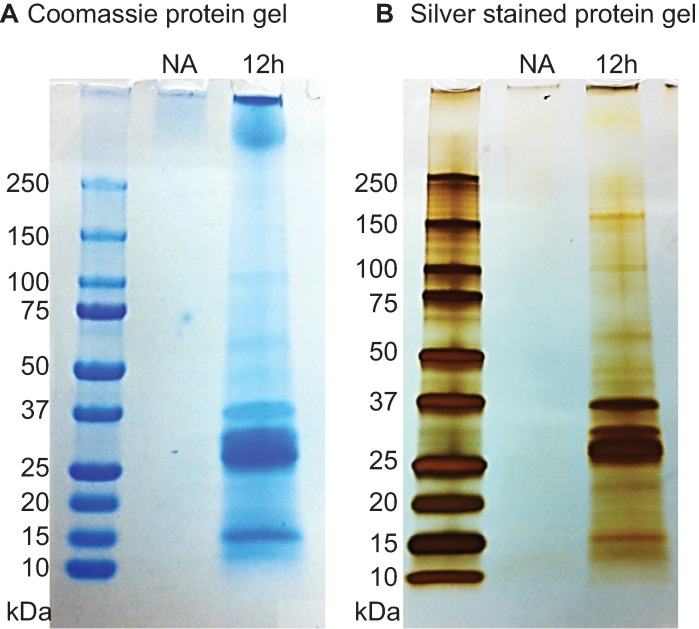
Protein secretion of activated and non-activated *S*. *scapterisci* IJs. Protein gels showing the quantity and diversity of proteins secreted by activated (12h) and non-activated (NA) IJs. Equal percentage of total volumes of concentrated proteins from non-activated and activated *S*. *scapterisci* IJs was loaded onto each gel. IJs were activated with 50% cricket homogenate. **A.** A protein gel stained with colloidal Coomassie Blue. **B.** A protein gel with silver staining.

Naïve or non-activated *S*. *scapterisci* IJs are usually ensheathed in the L2 cuticle with their mouths sealed in a non-feeding, stress-tolerant state [36]. We found that non-activated IJs secrete very little protein (Fig 4). When IJs become activated for 12 hours, they secreted a significantly higher amount of proteins and the secretion is a complex mixture of many proteins distributed over a wide range of molecular weight (Fig 4). The most abundant proteins were between 25–37 kDa (Fig 4). We selected the 12-hour activation for venom collection because we were interested in studying secreted proteins at earlier stages during IJ activation and the total activation rate after 12 hours is more than 50% (Fig 3 and S1 File). These results suggest that the morphological features we have used to score IJ activation correlate with metabolic and biochemical changes that may be important for parasitism. After venom collection, we counted the activation rates and checked the quality of the nematodes. We rarely found damaged nematodes. However, we cannot rule out the possibility that some of the secreted proteins may have come from a small number of damaged nematodes. Further study is required to identify the proteins in order to distinguish secreted proteins from contaminating proteins.

### Selective activation of *S*. *scapterisci* IJs by cricket homogenate

**Fig 5:**
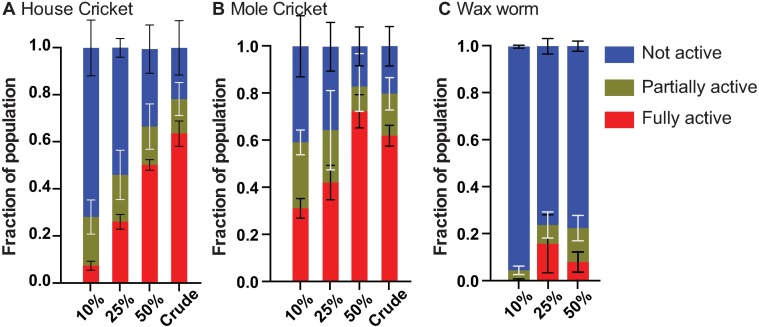
Concentration- and host-dependent activation of *S*. *scapterisci* IJs. IJs were exposed to three different types of insect homogenates (house crickets, mole crickets, and waxworms) at multiple concentrations (10%, 25%, 50%, and crude homogenates for both types of crickets and 10%, 25%, and 50% for waxworms). Three biological replicates of triplicate experiments were performed for each time point (n≥171 nematodes per replicate; n≥591 nematodes total per condition) (S2 File). **A.** Higher concentrations of house cricket homogenate resulted in significantly higher activation rates among IJs (p<0.0001; one-way ANOVA). 25%, 50%, and crude homogenate all produced significantly higher activation than 10% homogenate (p<0.0001; unpaired t test). 50% and crude homogenate both induced significantly higher activation than 25% homogenate (p<0.0001; unpaired t test), and crude homogenate induced significantly higher activation than 50% homogenate (p = 0.04; unpaired t test). **B.** Higher concentrations of mole cricket homogenate resulted in significantly higher activation rates among IJs (p<0.0001; one-way ANOVA). There was no significant difference in activation between 10% and 25% mole cricket homogenate, but 50% and crude homogenate both induced significantly higher activation than 10% or 25% homogenate (p≤0.03). **C.** Higher concentrations of waxworm homogenate resulted in significantly higher activation rates among IJs (p<0.0001; one-way ANOVA). 25% and 50% homogenate both induced significantly more activation than 10% homogenate (p≤0.002), but there was no significant difference between 25% and 50% waxworm homogenate. Comparing activation rates between the different hosts, mole cricket homogenate induced significantly higher activation than house cricket homogenate at 10% (p<0.0001; unpaired t test), and 50% (p = 0.009; unpaired t test). Both cricket homogenates induced significantly higher activation in *S*. *scapterisci* IJs than waxworm homogenate (p<0.0001; unpaired t test).

We used homogenates from three different insect hosts, *A*. *domesticus* house crickets, *S*. *borellii* mole crickets, and *G*. *mellonella* waxworms, to test their effect on *S*. *scapterisci* IJ activation. We also tested the influence of host homogenate concentrations (10%, 25%, 50%, and crude homogenate) on IJ activation. We were unable to test crude waxworm homogenate since it was too thick and contained a large amount of debris making it difficult to obtain clean IJs for microscopic observation. We selected 18 hours of exposure for this assay because the expansion of the pharynx is more obvious in fully activated nematodes. We found that higher concentrations of all three homogenates resulted in significantly higher IJ activation rates (Fig 5A and S2 File) (p<0.0001). The ratio of partially activated IJs was significantly different between concentrations of mole cricket homogenate and between concentrations of waxworm homogenate (p = 0.03 and p = 0.0004 respectively) but not for house cricket treatments (p = 0.4). Exposing IJs to high concentrations of homogenate made subsequent use of the activated IJs quite difficult, since they were coated with insect tissue. Using lower concentrations of homogenate, such as 25%, may be preferable for some downstream applications.

Comparing activation rates for each type of host homogenate we found that for house cricket homogenate, 25%, 50%, and crude homogenate all produced significantly higher activation than 10% homogenate (p<0.0001) (Fig 5A and S2 File). 50% and crude house cricket homogenate both induced significantly higher activation than 25% homogenate (p<0.0001), and crude homogenate induced significantly higher activation than 50% homogenate (p = 0.04). Using mole cricket homogenate, there was no significant difference in activation between 10% and 25% homogenate, but 50% and crude homogenate both induced significantly higher activation than 10% or 25% homogenate (p≤0.03) (Fig 5B and S2 File). There was no significant difference between 50% and crude homogenate. Using waxworm homogenate, 25% and 50% homogenate both induced significantly more activation than 10% homogenate (p≤0.002) (Fig 5C), but there was no significant difference between 25% and 50% waxworm homogenate.

Comparing activation rates of IJs exposed to different insect homogenates, mole cricket homogenate induced significantly higher activation than house cricket homogenate at 10% (p<0.0001), and 50% (p = 0.009) concentrations. Both cricket homogenates induced significantly higher activation in *S*. *scapterisci* IJs than waxworm homogenate (p<0.0001).

## Discussion

*Steinernema scapterisci* is an unusual member of the entomopathogenic nematode guild. It has been described as a cricket specialist but the nature of specialization is poorly understood. Several questions have arisen regarding the specialization of *S*. *scaptersci*, such as the role of host-seeking behavior in specialization and limitations in the IJs ability to infect and kill certain hosts. Previous literature suggests it is not host-seeking behavior that determines *S*. *scapterisci* specialization; *S*. *scapterisci* IJs are attracted to crickets, waxworms, and other potential hosts and this attraction is temperature-dependent [3, 37]. It has also been shown that *S*. *scapterisci* IJs are capable of infecting and reproducing in *G*. *mellonella* [12, 15], but has reduced efficacy compared to other steinernematids, suggesting that the host specialization occurs after IJs enter the host and is context-dependent.

Our research goal was to understand the biology underlying the specialization of *S*. *scapterisci* as a parasite of crickets. We began by examining how *S*. *scapterisci* IJs recover from the developmentally arrested infective juvenile state and resume development. This process is similar to dauer recovery in *Caenorhabditis elegans* [38]; however, EPN dauer recovery entails the active expulsion of pathogenic bacteria and likely includes the active secretion of pathogenic products by the nematode [39, 40]. Therefore we refer to EPN recovery from dauer as IJ activation. Previous studies have shown that exposure to host cuticle leads to increased attraction of IJs to host volatiles, suggesting a kind of behavioral activation in *S*. *carpocapsae* [41, 42]. Here we studied the physiological activation of IJs as they are exposed to insect homogenate.

To study IJ activation we first identified quantifiable morphologic changes that could be used to differentiate between activated and non-activated IJs (Fig 2). We found that by examining the state of the mouth, pharyngeal bulb, and anterior gut, we could reliably distinguish between non-activated, partially activated, and fully activated IJs (Fig 2). Some early-activated IJs developed to L4 and young adult stages when incubated for more than 18 hours in cricket homogenate and in this study we categorized those as fully activated IJs. In future studies it may be informative to distinguish between activated IJs and nematodes that have developed beyond the activated L3 stage.

Using these character states, we performed a time course of activation, exposing *S*. *scapterisci* IJs to house cricket homogenate for different amounts of time and were able to show that IJ activation occurs temporally (Fig 3). We found that maximal *S*. *scapterisci* IJ activation occurred after 18 hours. This activation correlated with the secretion of a diverse complement of proteins by the nematodes (Fig 4). *S*. *scapterisci* has been shown to be highly pathogenic to insect hosts and capable of reproducing *in vivo* without its symbiotic bacterium *Xenorhabdus innexi* [12, 17]. We suspect that its virulence may depend on the arsenal of secreted products that are induced upon IJ activation. Further research into the identity and function of these secreted products is warranted.

Although the original description of *S*. *scapterisci* suggested that it was extremely poor at infecting *G*. *mellonella* larvae, even noting that it was incapable of reproducing in waxworms [18, 26], subsequent studies showed that there was appreciable virulence of *S*. *scapterisci* to waxworms and that IJs would emerge from such infections [12, 15]. We examined IJs exposed to house cricket, mole cricket, and waxworm homogenate to test whether IJs activation was context-dependent. Our data support the description of *S*. *scapterisci* as a cricket specialist, with striking differential activation in cricket tissue and poor activation in waxworm tissue after 18 h of exposure (Fig 5). Our results indicate that *S*. *scapterisci* has evolved to activate when detecting signals rich in cricket tissue.

Entomopathogenic nematodes are defined by their ability to kill hosts quickly and their use of bacteria in infection and reproduction [10, 43]. We imagine that an EPN lineage could evolve independence from its bacterial symbiont, in which case it would still be an insect parasite, but it would cease to be an EPN. How might an EPN evolve independence from its bacterial symbiont? We postulate that over evolutionary time the nematode might become pathogenic on its own, able to kill hosts and liberate nutrients without relying on pathogenic bacteria. *S*. *scapterisci* has many of the traits of an EPN that may be on its way to evolving independence from its bacterial symbiont. It promiscuously associates with different bacteria [15], axenic strains display lethal infectivity [17], and it is reported to be capable of reproducing in insect hosts without any exogenous bacteria being present [12]. Coevolutionary theory suggests that specialist parasites such as *S*. *scapterisci* should have lower virulence and a tighter association with its bacterial symbiont than generalist parasites such as *S*. *carpocapsae* [28, 29], however our current understanding of *S*. *scapterisci* does not fit these predictions. *S*. *scapterisci* is a unique, understudied EPN with the potential to be a powerful comparative model for studying specialization, host-parasite interactions, and the evolution of symbiosis, among other biology. We have described an improved method for efficient cultivation of *S*. *scapterisci in vivo* using house crickets. We have developed a method for quantifying IJ activation and harvesting the secreted proteins. Our data support the description of *S*. *scapterisci* as a cricket specialist and these improved methods may serve as a foundation for additional studies of this parasitic nematode.

## Supporting Information

S1 File Activation data from the time course experiments. (XLSX)

S2 File Activation data using different insect homogenates and different concentrations of homogenate. (XLSX)
